# The Role of Inflammation and Inflammasome in Myeloproliferative Disease

**DOI:** 10.3390/jcm9082334

**Published:** 2020-07-22

**Authors:** Lucia Longhitano, Giovanni Li Volti, Cesarina Giallongo, Mariarita Spampinato, Ignazio Barbagallo, Michelino Di Rosa, Alessandra Romano, Roberto Avola, Daniele Tibullo, Giuseppe Alberto Palumbo

**Affiliations:** 1 Section of Biochemistry, Department of Biomedical and Biotechnological Sciences, University of Catania, 95123 Catania, Italy; lucia.longhitano@unict.it (L.L.); livolti@unict.it (G.L.V.); mariaritaspampinato93@gmail.com (M.S.); ravola@unict.it (R.A.); 2 Department of Scienze Mediche Chirurgiche e Tecnologie Avanzate “G.F. Ingrassia”, University of Catania, 95123 Catania, Italy; palumbo.ga@gmail.com; 3 Section of Biochemistry, Department of Drug Sciences, University of Catania, 95123 Catania, Italy; ignazio.barbagallo@unict.it; 4 Section of Human Anatomy, Department of Biomedical and Biotechnological Sciences, University of Catania, 95123 Catania, Italy; mdirosa@unict.it; 5 Division of Hematology, Department of General Surgery and Medical-Surgical Specialties, A.O.U. “Policlinico-Vittorio Emanuele”, University of Catania, 95123 Catania, Italy; sandrina.romano@gmail.com

**Keywords:** myeloproliferative neoplasms (MPNs), myelofibrosis (MF), polycythemia vera (PV), essential thrombocythemia (ET), inflammasome, inflammation

## Abstract

Polycythemia vera (PV), essential thrombocythemia (ET) and primary myelofibrosis (PMF) are rare hematological conditions known as myeloproliferative neoplasms (MPNs). They are characterized for being *BCR-ABL* negative malignancies and affected patients often present with symptoms which can significantly impact their quality of life. MPNs are characterized by a clonal proliferation of an abnormal hematopoietic stem/progenitor cell. In MPNs; cells of all myeloid lineages; including those involved in the immune and inflammatory response; may belong to the malignant clone thus leading to an altered immune response and an overexpression of cytokines and inflammatory receptors; further worsening chronic inflammation. Many of these cytokines; in particular, IL-1β and IL-18; are released in active form by activating the inflammasome complexes which in turn mediate the inflammatory process. Despite this; little is known about the functional effects of stem cell-driven inflammasome signaling in MPN pathogenesis. In this review we focused on the role of inflammatory pathway and inflammasome in MPN diseases. A better understanding of the inflammatory-state-driving MPNs and of the role of the inflammasome may provide new insights on possible therapeutic strategies

## 1. Introduction

Myeloproliferative neoplasms (MPNs) are a closely related group of rare, but potentially life-threatening, diseases caused by hyperproliferation of bone marrow stem cells. In MPNs, the production of blood cells (hematopoiesis) in the bone marrow is defective, resulting in the production of an excessive number of blood cells. MPNs include three main conditions: polycythemia vera (PV), essential thrombocythemia (ET) and myelofibrosis (MF) [1], which are associated with frequent disease-related complications, such as venous and arterial thrombosis, hemorrhages and transformation to acute myeloid leukemia (AML) [2]. These neoplasms are characterized by a clonal proliferation of an abnormal hematopoietic stem/progenitor cell [3]. PV is characterized by an excess of erythrocytes and predominant erythroid lineage involvement and with a variable hyperplasia of the megakaryocytic/granulocytic lineages [3]. ET is characterized by an increased platelet count with a concomitant megakaryocytic hyperplasia, whereas PMF is a more heterogeneous disorder in terms of clinical and biologic characteristics, characterized by the presence of megakaryocytic hyperplasia and bone marrow fibrosis [3]. The MPNs are defined by a chronic inflammatory status contributing to microenvironmental transformation necessary for supporting tumor progression and severity of the disease [4]. inflammation plays an important and defined role in cancer development affecting all tumorigenesis stage, including initiation, promotion, malignant conversion, invasion and metastasis [5]. Indeed, cancer-related inflammation is triggered by a series of signals from immune system cells such as macrophages, dendritic cells (DCs), NK cells (natural killer), neutrophils and T and B lymphocytes. Several studies showed that some genes involved in inflammasome activation, were significantly overexpressed also in MPNs [6,7,8]. Therefore, MPN represents a useful model to assess the possible relationship between clonal development of a hematologic malignancy and chronic inflammation [1]. Chronic inflammation has been considered for long time a key element in the development of MPNs, which are maintained by a continuous release of proinflammatory cytokines, chemokines [9] and ROS accumulation leading to genetic instability and successively to the development of neoplasms and their progression [10,11,12]. In the inflammatory immune response, the activation of inflammasome is a key event that plays a dual role in cancer development and progression. The role of inflammasome in cancer growth and progression is still controversial and several lines of evidence showed different effects in various cancer types; therefore, the aim of the present review is to describe the possible relationship between inflammasome, related cytokines and myeloproliferative neoplasms.

### Inflammasome

**Figure 1:**
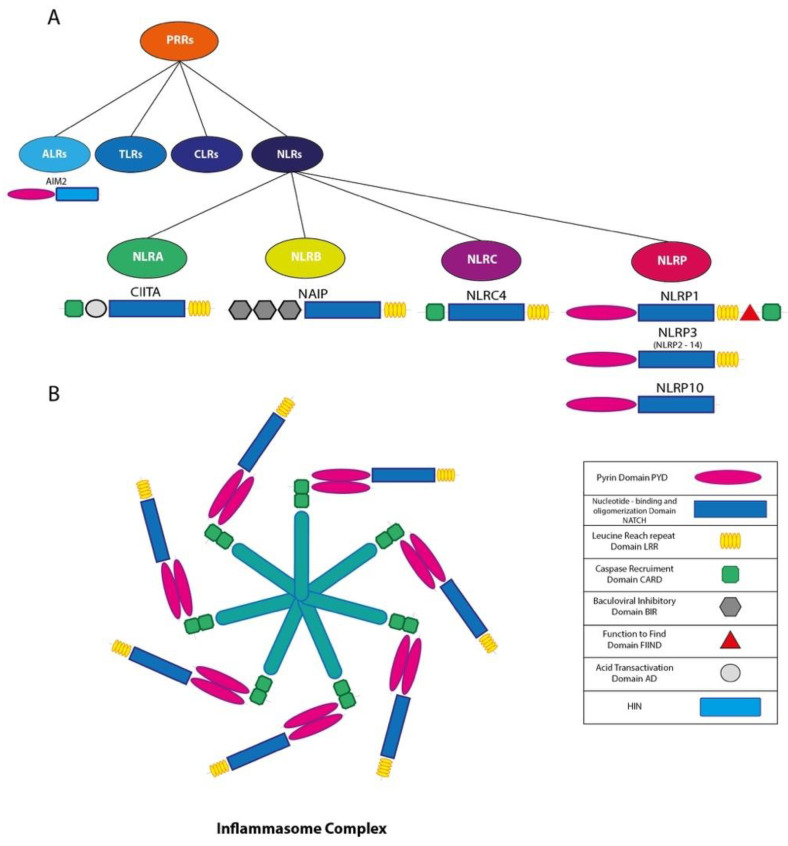
(**A**) Schematic representation of Pattern Recognition receptors (PRR) and nucleotide-binding oligomerization domain (NOD)-like receptors (NLRs); (**B**) schematic representation of inflammasome complex.

The term inflammasome was firstly used by Tschoppand co-workers in 2002 [13] when describing a high-molecular-weight cytosolic complex in stimulated immune cells responsible for the activation of the inflammatory caspase-1. inflammasomes are indispensable mediators of the innate immune response to infection [13]; they are constituted of large multimeric intracellular complexes that are capable of controlling activation of the proteolytic enzyme caspase-1 [13], which in turn regulates the proteolytic maturation of Interleukin-1β (IL-1β) and Interleukin-18 (IL-18), as well as a rapid, noxious, inflammatory form of cell death termed pyroptosis through cleavage of gasdermin D (GSDMD) [14,15]. Activation of inflammasome leads to an inflammatory response resulting either anti- and/or pro-tumor growth. inflammasomes are generally formed of a pattern recognition receptor (PRR), an apoptosis-associated speck-like protein (ASC) and the cysteine protease caspase-1 [16]. Five different families of PRRs have been described so far including nucleotide-binding oligomerization domain (NOD)-like receptors (NLRs), absent in melanoma 2 (AIM2)-like receptors (ALRs), Toll-like receptors (TLRs), Rig-I-like receptors (RLRs) and C-type lectin receptors (CLRs) [17,18]. Previous data suggest that other NLR and ALR family proteins (i.e., NLRP6, NLRP9b and pyrin) may also form functional inflammasomes [19,20,21,22] (Figure 1A). Assembly of inflammasome complexes is triggered by cytosolic sensing of pathogen-associated molecular patterns (PAMPs) or host-derived signals associated with cell stress (danger-associated molecular patterns, DAMPs) [23,24]. All together these complex series of biochemical events lead to the activation of the inflammasome and induce an immune response inhibiting pathogen replication [23,25,26,27] (Figure 1B). Intracellular danger signals (danger-associated molecular patterns) released from damaged or dying cells are also able to activate inflammasomes, but impaired inflammasome signaling causes and hyperinflammatory state, leading to the development of autoimmune and neurodegenerative diseases and cancer progression [28,29,30,31,32,33].

**Figure 2:**
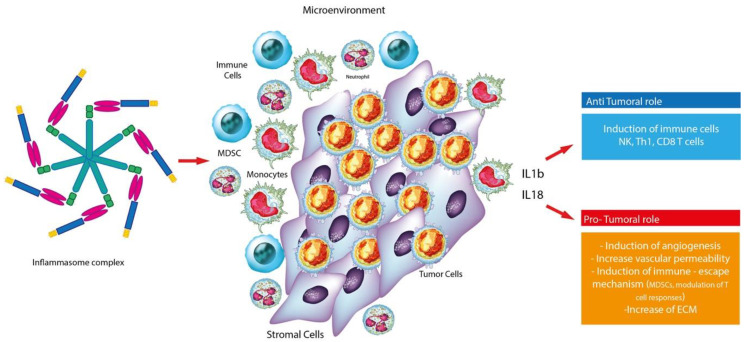
Graphic representation of the dual role of the inflammasome complex in cancer.

Several studies showed that aberrant inflammasome activation is often associated with the development and progression of cancer. In particular, tumor associated inflammation is triggered by a variety of immune cells, including neutrophils, T and B lymphocytes, macrophages, NK cells and DC cells. Other studies indicate that inflammasome may have ambivalent effect in tumors: it can contribute to the pathogenesis, development and neoplastic progression and maintenance of the tumor microenvironment or on the contrary suppress tumor growth through the phenomenon of pyroptosis and death of pre-malignant cells. Such different effect can in part be explained by the heterogenicity of cancer cells and the fact that different inflammasomes have different roles. [34,35,36,37]. Consistently, mutations in genes encoding inflammasome components often lead to susceptibility to cancer, infection and autoinflammatory diseases in humans [35,38,39,40,41,42,43,44,45]. Taken together, these results suggest that the pro-tumorigenic or antitumorigenic properties of inflammasomes and related cytokines are largely determined by the types of cells, tissues and organs involved (Figure 2).

## 2. Inflammation and Myeloproliferative Neoplasms (MPNs)

### 2.1. Mutation in MPNs

MPNs are characterized by abnormal proliferation of bone marrow stem cells accompanied by changes in the number of platelets, red blood cells and white blood cells; PMF also presents with an increase in bone marrow fibrosis, splenomegaly and extra-medullary hematopoiesis (EMH). EMH is observed not only in the liver and spleen, but also in the lymph nodes, in the urogenital system, in the serum surfaces and in the epidural and paraspinal spaces because of modification of the *CXCL12/CXCR4* axis [46]. In most cases MPNs are secondary to genetic defects regarding pluripotent stem cell populations leading to cell proliferation during the disease [47]. Such genetic defects mainly concern mutations involving *MPL*, *JAK2* and more recently, *CALR* genes, which are involved in the *JAK/STAT* pathway with its consequent dysregulation [48]. Altogether these mechanisms induce the abnormal signaling of *STAT* transcription factors with consequent cell growth and proliferation. *STAT3*, in particular, is closely linked to the development of cancer through the activation of immunomodulatory cytokines (IL-6, IL-10 and IL-17), growth factors (FGF, VEGF) and matrix metalloproteinases [49]. *STAT3* deletion result in lower white blood counts, lower spleen weights and a reduced degree of reticulin fibrosis, reduced disease severity and cytokine-mediated inflammation, similar to the effects observed with ruxolitinib therapy [50]. *JAK2^V617F^* is the best characterized mutations observed in these disorders with a prevalence of more than 95% in PV, 50%–70% in ET and 40%–50% in PMF [48,51]. *JAK2^V617F^* mutation negative cases of PV may present *JAK2* mutations in exon 12 present at a frequency of approximately 2%–3% [48,52]. *JAK2^V617F^* negative cases of ET and PMF can contain *MPL* mutations occurring at the frequency of 5% to 10% [48,53]. As far as concern *CALR* mutation, this was found to occur in most MPN patients with non-mutated *JAK2* or *MPL* [48,53,54]. Although *JAK2* is the most frequent mutation, there are patients without any of the three mutations and are therefore called triple negatives [48]. In investigations of large cohorts of MPN, *JAK2^V617F^* can be detected in 95% of patients with PV and *JAK2* exon12 mutation in the remaining 5% of patients [1]. Among ET patients, *JAK2^V617F^* can be detected in 60% and 65% of patients, *MPL^W515 L/K^* in approximately 5% and *CALR* mutation in approximately 20% and 25%. Very recently, several other mutations have been associated with MPN [48], including *TET2, ASXL1, IDH1/2* and *SRSF2* [55]. Regardless of the molecular state, all patients present a deregulation in *JAK/STAT* signaling. In all MPNs, megakaryocytes proliferate, acquire multilobulate nuclei and exhibit clustering in the bone marrow [56]. These cells are characterized by an abnormal localization of P selectin in their intracytoplasmic vacuoles and in the membrane demarcation system (DMS) which leads to an increased emperipolesis of neutrophils which release their enzymes into megakaryocytes and thus releasing other cytokines such as the transformation of the growth factor beta (TGF-β), platelet-derived growth factor (PDGF) and fibroblast growth factor (FGF) from their alpha granules [57].

### 2.2. Treatment in MPNs

Despite therapies targeting clones that support myeloproliferation, PMF is still considered an incurable disease, except for patients who successfully undergo an allogeneic stem cell transplantation [58]. To date, the only effective pharmacological therapy includes JAK inhibitors (JAKi). The prototypical compound of this class is ruxolitinib. However, while ruxolitinib is able to provide significant improvements in splenomegaly, associated clinical manifestations and constitutional symptoms related to the disease, at least part of its clinical benefits have been associated with a marked downregulation in serum proinflammatory cytokines [59] produced in particular by immunological and hematopoietic cells, both after four weeks of treatment [60] and after 24 months [61] demonstrating the importance of inflammation in the pathologic process [62].

### 2.3. Inflammation in MPNs

**Figure 3:**
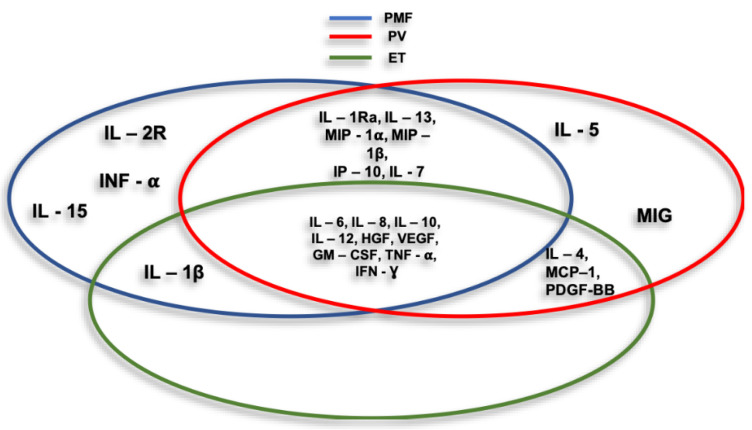
Cycles represent cytokines and chemokines associated with myeloproliferative neoplasms (MPNs) main entities.

In recent years it has emerged that the tumor microenvironment (TME) and in particular the inflammatory microenvironment [63], plays a fundamental role in the pathogenesis of MPN. This progressively transformed tumor microenvironment (TME) is infiltrated by different types of immune cells that are likely to kill cancer cells in the initial stages, but over time the inability of immune cells to inhibit the abnormal growth of cancer cells prevails depending on a variety of events occurring at the tumor site [36]. Hence, MPNs, that are initiated by the appearance of a myelostimulatory mutation, propagate through an evolving cascade of inflammatory conduits that entail dramatic symptoms and impairment of the quality of life of affected patients [47]. Taken all together these evidences suggest that MPN is as a chronic tumor model driven by inflammation [64], in which chronic inflammation plays a critical role in the development and maintenance of MPN itself [49]. Consistently, numerous studies have been carried out over the years confirmed this theory and it is now clear that there is a close relationship between chronic inflammation and the pathogenesis of MPN. An evaluation of abnormal cytokine expression determined that primary myelofibrosis (PMF) patients had significantly increased levels of IL-1 β, IL-1RA, IL-2R, IL-6, IL-8, IL-10, IL-12, IL-13, IL-15, TNF-alpha, G-CSF, IFN-α, MIP-1α, HGF, IFN-γ and VEGF in addition to reduced IFN-γ levels while MIP-1β gave conflicting results. In PV, patients increased levels of IL-1RA, IL-4, IL-5, IL-6, IL-7, IL-8, IL-10, IL-12, IL- 13, IFN-γ, GM-CSF, MCP-1, MIP-1α, MIP-1β, HGF, IP-10, MIG, MCP-1, PDGF, TNF-α, IFN-γ and VEGF were measured [65,66,67]. An analysis of ET patients reported the presence of elevated levels of IL-1 β, IL-4, IL-6, IL-8, IL-10, IL-12, HGF, GM-CSF, IFN-γ, MCP-1, PDGF, TNF-α and VEGF. Taken together this evidenceline, levels of reported cytokines were higher compared to healthy controls, however some of these differences were not significant [65]. Interestingly, IL-4, IL-8, GM- CSF, IFN-γ, MCP-1, PDGF and VEGF appeared to be significantly higher in ET patients when compared to PV populations and may be used as markers to distinguish the two disorders [67] (Figure 3).

Some studies showed that among the myriad of cytokines evaluated in the MPN, could be prognostic for patients [64], such us IL-1 β which mediates inflammation both at tissue and systemic level; IL-6 that induces the activation of *JAK/STAT* pathway; IL-8 that influences the tumor microenvironment by retrieving MDSCs; VEGF which promotes angiogenesis; TGF-β which in primary myelofibrosis is associated with bone marrow fibrosis. In healthy subjects the inflammatory cascade is driven by the interaction finely regulated by cellular responses and stimulating factors/cytokines. Dysregulation of this balance can lead to chronic inflammation, which is characteristic of several pathologies including MPNs. An Italian study, conducted by Sollazzo et al. showed significantly higher IL-1β levels in PMF patients than healthy controls and observed an increase in the proliferation of CD34+ cells treated with culture media containing IL-1 *β*, suggesting that IL-1 *β* plays an important role in the progression of myelofibrosis [63]. In MF, the combination of TNF-*α* and TIMP-1 has been shown to promote survival of CD34+ stem cells, whereas the combination of ATP and TNF-α has been shown to reduce proliferation [68]. Previous reports showed that *JAK2^V617F^* positive patients have significantly higher levels of IL-8, IL1B, IL-17A and IFN versus triple-negative (*JAK2, MPL* negative) patients [69]. TGF-β, a cytokine involved in normal hematopoiesis and in hematological malignancies, plays a key role in the progression of MPNs, so many studies have focused on the its role. Campanelli et al. showed, through CCL64 cell line tests, that PV, ET and PMF patients had significantly higher levels of TGF-*β* both in peripheral blood (PB) and bone marrow compared to healthy controls [70]. In addition to myeloproliferation, PMF is characterized by bone marrow fibrosis, neoangiogenesis and osteosclerosis [58]. Megakaryocytes and monocytes derived from malignant clones produce high levels of TGF- *β* other than PDGF, FGF and VEGF. In particular, TGF- *β* exerts pro-fibrotic effects on fibroblasts and is considered to be the main driver of fibrosis, as is able to induce collagen production. The inhibition of TGF- *β* in different experimental models resulted in a reduction of fibrosis [71].

### 2.4. Inflammasomes and Myeloproliferative Neoplasms

As described above, chronic inflammation is considered a driving force for the development of MPN, intensified by the continuous release of pro-inflammatory cytokines and chemokines that in the aging bone marrow niche can predispose patients to leukemic transformation. Many of these cytokines, in particular IL-1 β and IL-18, are released in active form by activating the inflammasome complexes which in turn mediate the inflammatory process. Despite this, little is known about the functional effects of stem cell-driven inflammasome signaling in MPN pathogenesis. Wang et al. showed that *JAK2^V617F^* positive macrophages expressed greater production of cytokines and chemokines has been demonstrated in CD11+ splenic cells, which in turn has led to an increase in the secretion of IL-1 *β* and an increase in plasma levels of IL-18, which could contribute to increasing the production and activation of neutrophils and the entry of leukocytes into the lesion [7]. Another study by Shinar et al. has shown that a mutation in the Mediterranean fever gene (MEFV), highly expressed in myeloid cells and coding for pyrin, a cytoplasmic protein that regulates the maturation and secretion of the pro-inflammatory cytokines IL-1β and IL-18 in the complex of the inflammasome, resulted in Mediterranean fever disease in *JAK2^V617F^* patient post-polycythemia myelofibrosis (PPV-MF) [72]. The microarray analysis conducted by Liew et al. identified numerous genes involved in the activation of the inflammasome, such as AIM2, IL-1 β, CASP1, which were significantly upregulated in the cells where *JAK2^V617F^* was induced. In particular, induction of *JAK2^V617F^* leads to a significant inflammatory response consistent with recent studies demonstrating the involvement of IL-1*β* in the development of myelofibrosis in a *JAK2^V617F^* mouse model. Furthermore, these evidences further suggest that AIM2 is a downstream target of *JAK2^V617F^* in D9 cell line [6]. Given the crucial role of AIM2 in combination with CASP1, in converting pro-IL1B to its active form [73,74], the induction of the *AIM2*, *CASP1* and *IL1B* mRNAs in *JAK2^V617F^*-induced cells suggests that *IL1B* activation is linked to MPN development. Consistently, *JAK2^V617F^*-positive hematopoietic stem cells secrete IL1*β* inducing inflammation and promoting bone marrow myelofibrosis also in animal models [75].

## 3. Conclusions

Available data are not sufficient to fully establish the role of inflammasome in myeloproliferative neoplasms (MPN) and further studies are needed in order to clarify such a role. However, it was shown that there is an inflammatory-state-driving MPNs and that the available drugs (i.e., JAKi), are effective and improve symptoms through the immune system regulation although they do not present curative potential when used as a single agent. A better understanding on the role of the inflammasome may provide new insights on possible therapeutic strategies.
